# UVB induced reactivation leads to HSV1 in the corneas of virtually all latently infected mice and requires STING to develop corneal disease

**DOI:** 10.1038/s41598-024-52597-0

**Published:** 2024-03-21

**Authors:** Xiao-Tang Yin, Alexis Hartman, Nadia Sirajuddin, Deepak Shukla, Anthony St. Leger, Tammie L. Keadle, Patrick M. Stuart

**Affiliations:** 1 Department of Ophthalmology, Saint Louis University School of Medicine, St. Louis, MO USA; 2 Department of Ophthalmology & Visual Sciences, University of Illinois at Chicago, Chicago, IL USA; 3 Department of Microbiology and Immunology, University of Illinois at Chicago, Chicago, IL USA; 4 Department of Ophthalmology, University of Pittsburgh, Pittsburgh, PA USA; 5 Department of Biology, Washington University, St. Louis, MO USA; 6 Washington University, 7569 Amherst Avenue, University City, MO 63130-2805 USA

**Keywords:** Immunology, Microbiology, Diseases

## Abstract

Reactivation of latent herpes simplex type 1 results in virus returning to the cornea leading to recurrent herpetic stromal keratitis (rHSK). We compare two competing models to reactivate viruses from latency, UV-B irradiation and cyclophosphamide (CP). Results revealed that while both result in corneal recrudescence, only UV-B irradiation results in rHSK. To better understand the dynamics of reactivation, we analyzed corneas for both the presence of infectious viruses and the dynamics of exposure to multiple reactivations using UV-B. We noted that multiple reactivations result in progressively worse corneal disease. We also noted that expression of IFNα and STING, surragate markers for the presence of virus, are induced by the presence of reactivated virus. Studies to determine the importance of STING to the development of HSK revealed that in the absence of STING, mice do not develop significant HSK and the magnitude of the infiltrate of CD45+ cells in these corneas is significantly reduced. The resulting paucity of CD45+CD11b+GR-1+F4/80-neutrophils, and to a lesser extent CD45+CD11b+GR-1-F4/80+ macrophages in B6-STING KO mice following reactivation is likely the underlying cause for lack of rHSK as has been noted by ourselves and others. These results underscore the critical importance of STING’s role in developing rHSK.

## Introduction

Herpes simplex virus 1 (HSV-1) can infect the cornea of humans and cause herpetic stromal keratitis (HSK), which is a leading infectious cause of corneal blindness in the United States of America^1–5^. Although the immune mechanisms are not fully understood, HSK is an immunoinflammatory disease triggered by reactivation of HSV-1 that has traveled from the latently infected trigeminal ganglia to the eye where it produces disease by reactivating an inflammatory response in the eye against the virus^6^. Therefore, it is beneficial to conduct HSK experiments using recurrent models of disease in addition to those involving primary infection models, because recurrent models most resemble HSK disease in humans^7–9^. Primary and recurrent HSK have distinct mechanisms, because different cytokines and chemokines play different roles in each disease. For example, interleukin-6 plays a role in primary HSK but not recurrent HSK^10^. Clinically speaking, recurrent HSK mice models result in features different from primarily infected mouse models^7^. Just like HSK disease in humans, recurrent infections in mice result in microdendrites, focal stromal opacities, disciform endotheliitis, and corneal neovascularization^7,8^. Meanwhile, primary infections in mice result in a different clinical picture, unlike human disease, which includes multiple epithelial dendrites and diffuse stromal opacification^3^. Mice are not the only animals used to study recurrent HSK disease. Along with mice, dogs^11–13^ and rabbits^13–19^ have also been used to create HSV-1 reactivation models.

Due to economic and genetic benefits corresponding to using mice, mice have been the species of choice when studying both primary^3,6,7^ and recurrent models of HSK^8,9,11,12,20–25^. One of the early murine models of HSK used a modification of what is used in rabbits. In this model iontophoresis along with timolol was used, the addition of timolol being a key factor as this model displayed improved frequency of reactivation when compared to iontophoresis alone^20^. Other reactivation techniques used to reactivate latent infections in mice include using UV irradiation, hyperthermia, transcorneal epi iontophoresis, immunosuppressant treatment, and treatment with sodium butyrate in phosphate-balanced saline^8,9,11,12,21–25^. Approximately 24 h post thermal stress, mice treated with hyperthermia resulted in reactivation detected through harvested trigeminal ganglia^24^. The rest of the techniques mentioned above assessed reactivation by measuring the amount of viral shedding collected from eye swabs or eye washes^11,12,21–25^. Immunosuppressant and UV-B radiation resulted in a greater frequency of reactivation compared to other methods, which suggests UV-B irradiation and/or immunosuppressants may be the ideal technique of reactivation when designing a recurrent model to study HSK^26,27^.

The UV-B irradiation model has been used in many previous studies that have made important discoveries regarding HSK. Xia et al.^28^ utilized UV-B irradiation in experiments to demonstrate that elevated levels of interleukin-17 could be involved in the development of recurrent HSK^19^. Furthermore, the importance of LAT expression, as was reported in rabbits^13^, was also determined to be important in the UV-irradiation model in mice^9^. There are other studies pursuing the clinical potential of T-cell based immunotherapy or using CRISPR-Cas9 to cure recurrent HSK. These studies, which utilized a UV irradiation murine model, generated data suggesting the potential efficacy of such treatment in treating HSK^29^.

The current study was designed to investigate several issues. The first being a comparison of UV-B inducted and CP-induced reactivation. After demonstrating the superiority of the UV-B reactivation model over CP, we next performed studies to better determine what percentage of mice display reactivation following UV-B treatment of latently infected mice. These studies were followed by an analysis of the consequence of serial reactivations to corneal disease in individual mice. Finally, we discovered that STING expression was increased following reactivation, which led us to determine the role that STING expression plays in HSK.

## Materials and methods

### Mice

All procedures were carried out in accordance with ARRIVE Guidelines and the relevant guidelines and regulations for animal research. These investigations with mice conformed to the Association for Research in Vision and Ophthalmology (ARVO) Statement for the Use of Animals in Ophthalmic and Vision Research. These investigations, including experimental protocols and methods, were also reviewed and approved by the Saint Louis University Animal Studies Committee. C57BL/6 (B6) were purchased from NCI, C57BL/6J-*Sting1*^*gt*^/J (B6-Sting KO) were purchased from Jackson Labs and bred in our animal facility. NIH inbred strain of mice were originally obtained from Harlan OLAC (Oxford, England) and have been bred in the Saint Louis University animal facility for over 15 years under the supervision of the staff in Comparative Medicine.

### Infection of mice

For all experiments, except those shown in Table 1 (which used age matched female mice), we used age matched male and female mice from the aforementioned strains that were 6–10 weeks old. For recurrent disease, mice were typically infected on the scarified cornea with 10^6^ PFU HSV-1 McKrae strain as previously described^10^, unless otherwise indicated in the results section. Each mouse received an intraperitoneal (IP) injection of 0.5 ml pooled human serum (Sigma Chemicals, St. Louis MO; ED50 for virus neutralization = 1:1600) concurrent with infection. Administration of human anti-HSV antibodies at the time of ocular infection has been shown to protect mice from death and corneal disease during primary infection, while allowing for the establishment of latency and subsequent reactivation of virus after corneal UV-B exposure^8,10,21,26,27,30,31^. These human antibodies are undetectable at the time of UV-B irradiation, 5 weeks after primary infection. To confirm primary infection, eye swabs taken three days post-infection were evaluated for the presence of infectious virus. Typically, 100% of these infected mice were shedding virus at day 3^7^, should any mouse not demonstrate infectious virus, it would be eliminated from the study. Furthermore, it is rarely observed (less than 10%) that these mice will develop significant scarring from primary infection. Should they display such scarring, they are also eliminated from the study.

**Table 1:** Virological analysis of mice following UV-B and/or CP reactivation.

	UV-B	CP	UV-B + CP
% Positive swabs^a^	34	33	37
Peak titer (Log10)^b^	4.85 ± 0.32	5.15 ± 0.34	5.4 ± 0.43
Total shedding days^c^	21	19	24
Days shedding/mouse^d^	1.6 ± 0.14	1.7 ± 0.3	1.75 ± 0.35
Reactivation rate^e^	54%	50%	60%
Final day shedding^f^	Day 5	Day 6	Day 6
n	20	20	20

^a^The percent positive swabs is the percentage of virus-positive eye swabs (140 to 286 eye swabs per group) over the 10-day period following treatment of NIH mice.

^b^Peak viral titer, which occurred on Day 3 post-reactivation expressed as log_10_ plaque forming unit.

^c^Total shedding days number of days swab positive.

^d^Days shedding/mouse are the number of days that a positive mouse shed virus.

^e^Percent reactivation rate is the percentage of mice that reactivated.

^f^Final day shedding was the last day that a mouse was positive for a particular group. There were no significant differences between any of these treatments by students t-test.

### UV-B irradiation and virus reactivation

Mice were reactivated from latency as previously described^10,21^. Prior to reactivation, mice are swabbed to detect the presence of any virus in the tear film that might result from either persistence of virus in the cornea or spontanteous reactivation. It should be noted that viral positive mice are very rarely seen in wild-type mice, though some gene targeted mice do present with virus in their tears. Whenever an animal is shown to be positive prior to reactivation that animal is removed from further analysis. Briefly, the eyes of all latently infected mice were examined for corneal opacity before irradiation, and only animals with clear corneas were used. At least 5 weeks after primary infection, the eyes of latently infected and control mock-infected mice were exposed to 250 mJ/cm^2^ of UV-B light using a TM20 Chromato-Vu transilluminator (UVP, Inc., San Gabriel, CA), which emits UV-B at a peak wavelength of 302 nm. Irradiated mice were swabbed with sterile cotton applicators from day 0 to day 7, unless otherwise indicated. The day 0 swab was performed to eliminate any animal with infectious virus in their tear film prior to reactivation from consideration in that particular study. The swab material was cultured on Vero cells, as described^7^, to detect recurrent virus shedding from the cornea. Reactivation was defined as the finding of any HSV-positive eye swab on any day after UV-B exposure, with day 0 swabs serving as a control^21^. We also determined the result on HSK by subjecting mice to multiple reactivations by UV-B irradiation. This was accomplished by successively reactivating mice every 30 days at which point the clinical disease was resolving. Mice were observed for clinical disease following these reactivations and viral swabs taken for 7 days following each reactivation.

### Cyclophosphamide procedure

Mice that are latently infected with HSV-1 and control mice are injected with 150 mg/kg of cyclophosphamide (CP) (Sigma-Aldrich) on days 0, + 2, and + 4 intraperitoneally. CP was diluted in saline based on each individual animal’s weight. Mice were swabbed with sterile cotton applicators from day 0 to day 7 following CP treatment, unless otherwise indicated. The swab material was cultured on Vero cells, as described above, to detect recurrent virus shedding from the cornea. Reactivation was defined as the finding of any HSV-positive eye swab on any day after CP treatment, with day 0 swabs serving as a control^21^.

### Clinical evaluation

On the designated days after viral infection or UV-B reactivation, a masked observer, who is unaware of the experimental groups, examined the mouse eyes through a binocular dissecting microscope to score clinical disease. Stromal opacification was rated on a scale of from 0 to 4, where 0 indicates clear stroma, 1 indicates mild stromal opacification, 2 indicates moderate opacity with discernible iris features, 3 indicates dense opacity with the loss of defined iris detail except pupil margins, and 4 indicates total opacity with no posterior view. Corneal neovascularization was evaluated as described previously^10,21^ using a scale of from 0 to 8, where each of the four quadrants of the eye is evaluated for the presence of vessels that have grown into them. Corneal blink reflex was tested by loosely holding the mouse and touching the cornea with the blunt tips of surgical forceps without touching the eyelashes and whiskers. The cornea was divided into 5 areas (4 quadrants and center area), loss of blink reflex referred to the inability of the mouse to blink when an area was touched and was recorded as 0. Positive blink reflex referred to the ability to blink when an area of the cornea was touched and was recorded as 1. The total score of the 5 areas would be the final score of corneal blink reflex for a mouse. A score of 0 indicated a complete loss of corneal sensation such that the mouse failed to blink when any area of the cornea was touched. A score of 5 indicated retention of some degree of sensation such that the mouse blinked when any area of the cornea was touched^30,31^. Note: Uninfected, UV-B irradiated control mice were used as a baseline for any effects due to UV-B irradiation.

### Plaque assays for virus in the cornea

Mice were irradiated with UV-B and sacrificed on Day 3 and whole eyes removed to determine the presence of virus within the corneal samples. Eyes were frozen in Eppendorf tubes on dry ice a placed stored at – 80 °C until use. Eyes were thawed by adding 50 µl media to each tube and the eye placed on a sterile Petri dish to remove cornea with disinfected scissors and forceps along the limbus under microscope. The corneas were then ground 30–40 times using a plastic pestle, following with addition of 400 µl media. At this point the ground material was subjected to sonication using a Branson Sonifier 450 (Marshall Scientific, Hampton, NH) each cornea was subjected to a 30 s pulse followed by 15 s rest and another 30 s pulse on ice to control for any heat generation. The sonicate is then plated on VERO cells or placed at 4 °C until plated. Titers for each cornea were determined by plaque assay.

### RNA isolation

Mice were euthanized and eyes were frozen on dry ice and stored at − 80 °C. Upon thawing, 1 ml of TRIzol reagent (Life Technology) was added to each individual eye. The cornea was removed and ground 30 to 40 times to homogenize it. This was followed by addition of 200 µl chloroform and allowed to sit for 2–3 min at which time it was centrifuged at 12,000×*g* for 15 min at 4 °C. The top 0.4 ml of the aqueous phase is collected and transferred to a fresh tube to which 400 µl 70% alcohol was added and then applied to a spin cartridge from PuireLink RNA mini kit (Thermo Fisher Scientific). These cartridges were centrifuged at 12,000×*g* for 15 min washed with buffer and then buffer with ethanol. Finally, the RNA is eluted with 30 µl RNase-free water by incubating for 1 min at RT and then centrifuging the cartridge for 2 min at 12,000*g* to elute RNA. Store at − 80 °C.

### Detection of ICP4 lytic gene expression

Individual corneas were removed from female mice and placed in separate tubes, treated with DNase to remove DNA, and RNA was isolated. Once purified, the RNA it was reverse-transcribed using a High-Capacity RNA-to-cDNA kit (Applied Biosystems). We next performed the qPCR using a CFX96 Optics module (Bio-Rad Laboratories), operated by CFX Manager Software version 3.1, using ready-made Power SYBR Green PCR Master Mix (Applied Biosysytems) according to the manufacturer’s protocol. For ICP4 mRNA, the forward primer was 5′-GCG TCG AGG TCG T-3′ and the reverse primer was 5′-CGC GGA GAC GGA G-3′. Gene expression of each cornea was quantified relative to the expression level of GAPDH (Forward primer: 5′-ACTCCACTCACGGCAAATTC-3′ and reverse primer: 5′-TCTCCATGGTGGTGAAGACA-3′). We determined that GAPDH message could be reliably detected between 22 and 25 cycles of PCR expansion. Positive expression of ICP4 required between 25 and 28 cycles of PCR expansion. Any product detected above 32 cycles was deemed unreliable and thus considered negative.

### Western blot

Corneas were removed from mice at the indicated time points and stored at − 80 °C until use. For every 2 corneas, add 150 μL of ice-cold lysis buffer (Cell Signaling Technology, Danvers, MA) in addition of 1 mM PMSF (Cell Signaling Technology, Danvers, MA) rapidly to the tube, homogenize with a tissue grinder, then maintain constant agitation for 2 h at 4 °C (e.g., place on an orbital shaker in the fridge). Centrifuge for 20 min at 12,000 rpm at 4 °C in a microcentrifuge. Gently remove the tubes from the centrifuge and place on ice, aspirate the supernatant, and place in a fresh tube kept on ice; discard the pellet. Place samples in SDS loading buffer (Cell Signaling Technology, Danvers, MA) and boil for 5 min. Load equal amounts of sample into the wells of the 4–15% Mini-protean TGX stain- free SDS-PAGE gel (BioRad, Hercules, CA) along with a molecular weight marker (Cell Signaling Technology, Danvers, MA). Run gel for 1.5 h at 100 V. Transfer to nitrocellulose membrane. For staining, block with 5% BSA in Tris-buffered saline with 0.1% Tween^®^ 20 detergent (TBST). Incubate the membrane with 1:1000 dilutions of primary antibody (STING rabbit mAb [clone D1V5L], Cell Signaling Technology, Danvers, MA) in blocking buffer overnight at 4 °C. Wash with TBST and incubate with a 1: 3000 dilution of conjugated secondary antibody (anti-rabbit IgG, HRP-linked Ab) in blocking buffer at room temperature for 1 h. Wash with TBST and add substrate (SigalFire ECL Reagent A and B, Cell Signaling Technology, Danvers, MA) mixed in equal parts. Place the membrane into a transparent sheet then acquire image via chemidoc for chemiluminescence and normal image scanning methods for colorimetric detection. We also performed a loading control using a GAPDH antibody, clone 0411 (Santa Cruz Biotechnology).

### Immunofluorescence for IFN-α

Briefly, murine eyes were removed and placed in OCT compound (Sakura Finetek, Torrance, CA) and snap freeze. Multiple sections from 10 latently infected and reactivated mice were performed. Multiple sections were also taken from 4 latently infected and not reactivated mice, as well as multiple sections from 4 uninfected mice. The sections, (10 microns) and fixed with acetone at – 20 °C for 10 min and rinsed with cold PBS three times. The sections are then permeabilized by incubating with Triton-X 100 + 1% BSA + 10% goat serum (Sigma-Aldrich, St. Louis, MO) for 30–60 min at RT. We then removed blocking buffer and added rabbit anti-IFN-α serum (used at 1:50 PBL assay science, Piscataway, NJ) in fresh blocking buffer and incubated slides overnight at 4 °C. The slides were washed in TBST for 10 min three times at RT to remove unattached antibody and then incubated with AlexaFluor 488-conjugated anti-rabbit IgG (Thermo Fisher Scientific, Berkeley, CA) in PBS-T (0.05% Tween-20) for 1 h at RT. We then cover-slipped the slides with mounting fluid (ProLong gold antifade reagent with DAPI, Thermo Fisher Scientific, Berkeley, MO) and sections were analyzed by fluorescence microscopy with a Leica DFC345FX fluorescence microscope.

### Flow cytometric analysis

Cells were isolated from individual corneas as previously described^8,30^. Briefly, corneas were excised at defined time points and incubated in PBS-EDTA at 37 °C for 15 min at 37 °C. Stroma’s were separated from overlying epithelium and digested in 84 U collagenase type 1 (Sigma-Aldrich, St. Louis, MO) per cornea for 2 h at 37 °C and then were triturated to form a single-cell suspension. Suspensions were filtered through a 40-µm cell strainer cap (BD Labware, Bedford, MA) and washed and then stained with trypan blue to determine viability and to get a cell count of live cells. It should be noted that the viability of these cells did not vary significantly between the strains of mice used. Suspensions were initially stained with: PerCP-conjugated anti-CD45 (clone 30-F11, from BioLegend, San Diego, CA) and this was used to gate cells of bone marrow origin. For cornea suspension, cells were further stained with Alexa Fluor700-Gr-1 (clone RB6-8C5) and APC F4/80 (clone BM8) (from BioLegend, San Diego, CA); FITC conjugated anti-CD4 (clone RM4–5), PE-conjugated anti-CD8*α* (clone 53–6.7) (from PharMingen) and eFlour 660-conjugated CD11b (clone M1/70) (from eBiosciences, San Diego, CA). The samples were analyzed by the Microbiology flow cytometry core who did the gating and provided us with the data. The strategy we used for analysis was to initially gate on live cells by the and then the CD45^+^ cells. These CD45^+^ cells were further evaluated for T cell markers CD4 and CD8, or for macrophage markers F4/80^+^CD11b^+^GR-1^−^, or neutrophil markers GR-1^+^CD11b^+^F4/80^−^. Cells were then analyzed on a flow cytometer (FACSAria with FACSDIVA data analysis software; BD Biosciences).

### Statistics

All statistical analyses were performed with the aid of Sigma Stat for Windows, version 2.0 (Jandel, Corte Madera, CA). The log rank test was used to compare disease scores. Student’s unpaired *t*-test was used to compare virus titer data and flow cytometry numbers.

## Results

It has been reported that both CP and exposure to UV-B light leads to HSV-1 reactivation^32^. However, they have never been compared head-to-head for both their ability to reactivate or the resulting corneal disease which occurs following such treatments. Our laboratory compared CP with UV-B in a mouse strain, inbred female NIH mice, which has been shown to display the highest reactivation rates following UV-B irradiation^26,27^. Results indicate that the reactivation rate for CP treatment was very similar to that seen when UV-B irradiation was used (Table 1). When days shedding virus were compared, UV-B had a mean shedding of 4.1 days and CP, 2.3 days which was statistically significant (P = 0.014). It is interesting to note that these studies also tested the combination of CP + UV-B the result of which did not result in statistically greater numbers of mice shedding virus into the tear film (60%) than either UV-B (54%) or CP (50%) alone (Table 1). These data indicate that there is no additive effect of combined CP + UV-B treatment in stimulating virus release into the tear film.

**Figure 1:**
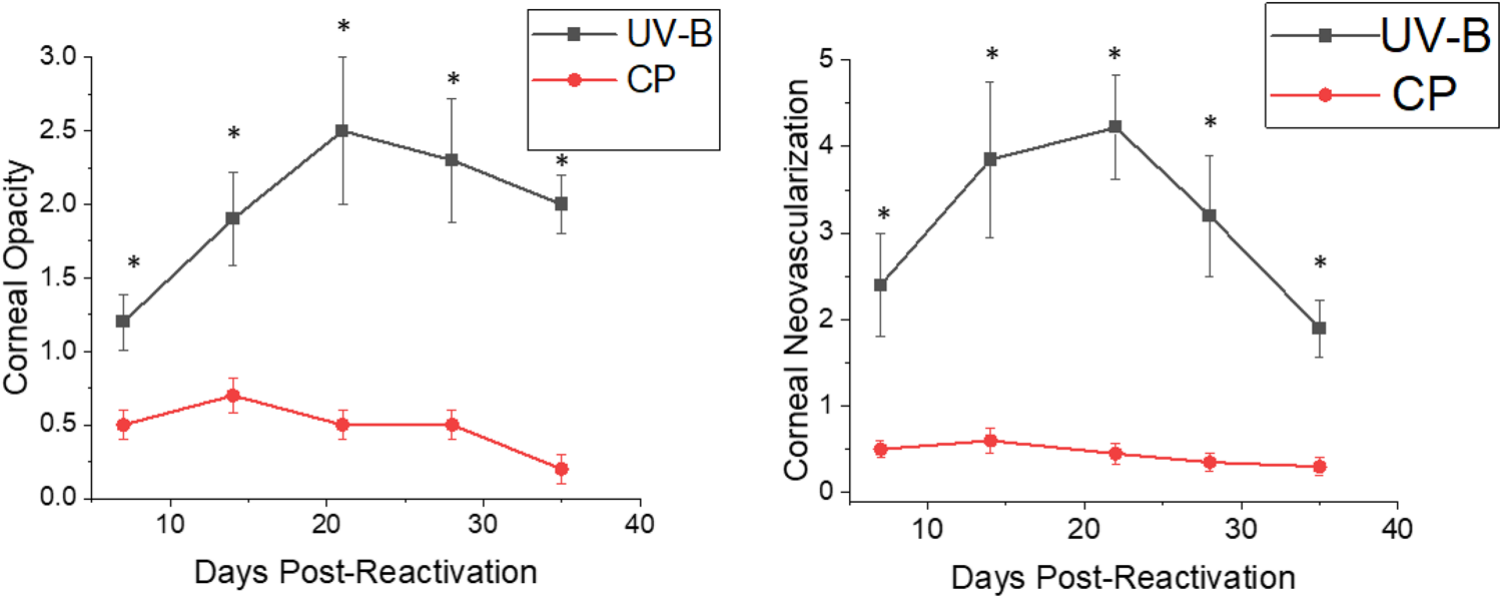
NIH mice displayed significantly greater recurrent HSK when reactivation was stimulated by UV-B irradiation compared to those stimulated with CP treatment. Eyes of mice were infected with 10^6^ pfu of HSV-1, McKrae strain. Six weeks following infection mice were either irradiated with UV-B irradiation or treated with CP to reactivate the latent infection. Each group consisted of 20 mice and were evaluated for corneal opacity or corneal neovascularization. Results are shown as mean ± SEM. P values for this comparison indicated statistical significance for all time points measured ranged from 0.01 to 0.001 by Rank Sum analysis (see asterisks). Similar data was observed from 3 independent studies, though the group sizes were smaller (data not shown).

When these mice were monitored for corneal disease following reactivation, the disease in CP treated mice was almost non-existent while UV-B reactivation resulted in significant viral-induced HSK disease (Fig. 1). We also monitored corneal disease in animals that were treated with both CP and UV-B irradiation, and such analysis indicated that these animals, like those treated with CP alone, did not develop significant corneal disease (data not shown), further indicating the immune suppression, which has been shown to have significant immunosuppressive effects^33^, likely does not allow for the development HSK.

As we evaluated these data, we were faced with the critical issue as to how well does shedding of virus into ocular tear film reflect actual number of mice that have virus in the corneas following UV-B reactivation. We had always believed that detection of viruses in tear film would be, at best, a reflection of the lower limit to how many mice are reactivating. To begin to better understand what a more accurate rate of reactivation would be we assayed several different parameters to determine the presence of virus in corneas following UV-B reactivation.

We have recently reported that viral antigen was expressed in virtually all corneas following reactivation^31^. While it is formally possible that the results shown in that publication^31^ could be due to persistent antigen in the cornea, we did not believe that to be the case. Nonetheless, we decided to remove corneas from latently infected female mice 3 days after UV-B reactivation grind them and then plate this tissue on Vero cells as a means of detecting intact infectious virus in reactivated corneas. The data from these extracts were then compared to latently infected mice that were not subject to UV-B reactivation. As shown in Table 2, less than 10% of mice that did not undergo UV-B treatment possessed an infectious virus in their corneas. The fact that the only mouse that displayed infectious virus in the absence of reactivation was a female mouse might suggest that female mice for whatever reason do not control latency as well as male mice. However, it should be pointed out that these mice did not display virus in their tear film. Furthermore, it was only one mouse and so it is not possible to make any significant interpretations from such a limited sample size. While almost all UV-B treated latently infected female mice (91%) demonstrated the presence of infectious virus (Table 2), the percent of male mice displaying infectious virus following UV-B treatment was 70%. While this difference may seem to be significant, upon statistical analysis it was not (P > 0.05). However, the difference in mean virus titer between female mice (913 ± 414) and male mice (20.6 ± 12)was significant (P < 0.05) (Table 2).

**Table 2:** Virological analysis latently infected undergoing UV-B induced reactivation and those not UV-B treatment.

Parameters	UV-reactivated	UV-reactivated	Latently infected	Latently infected
Measured	Female	Male	Female	Male
% Positive samples^a^	91%	70%	12%	0%
Mean ± SEM^b^	912.6 ± 414	20.6 ± 12	1.3 ± 0.9	0
Range^c^	0–6800 pfu	0–212 pfu	0–8	0
n^d^	23	24	6	6

^a^Extracts from NIH mice that possessed at least one pfu.

^b^Mean titer of virus ± standard error of the mean.

^c^Range of viral titers.

^d^Number of animals in group for this single experiment.

Statistical analysis indicates that there is no difference between female and male UV-reactivated mice for % positive samples. For other comparisons between these two groups, female mice displayed greater levels for mean (P < 0.05) and for Range (P < 0.05). Comparison between UV-reactivated and latently infected only reveal that the UV-reactivated mice were greater for all parameters measured (P < 0.005).

We next performed similar studies in which RNA was isolated from corneas derived from either latently infected mice that were subjected to UV-B treatment or those corneas from latently infected mice that were not exposed to UV-B treatment. We used the expression ICP4 as our target gene as it is critical in the ability of the virus to make infectious progeny^34^. Results from these studies indicated that only mice from latently infected mice that were UV-B reactivated possessed detectable viral RNA (4 of 9 mice), while none of the latently infected and unreactivated mice possessed detectible viral RNA (0 of 9 mice). However, the limitations of isolating viral RNA from corneal tissue indicates that it is very difficult to effectively isolate sufficient RNA for a full analysis. Nonetheless, we are confident that only corneas from reactivated mice have measurable RNA following UV-B reactivation.

We had previously hypothesized that multiple reactivations might lead to successively progressive corneal disease. Thus, we latently infected mice and reactivated them initially at 5 weeks following infection. These same mice were then subsequently reactivated 4 weeks following the first reactivation (Second); then 4 weeks following that reactivation (Third) and finally 4 weeks after that reactivation (Fourth). When we performed such an analysis, we found that our hypothesis was correct. Mice, even those without any corneal disease following their first exposure to UV-B irradiation, did upon subsequent reactivation stimulation developed significant corneal pathology (Table 3) when compared to naïve mice undergoing the same protocol of UV-B irradiation (Table 4).

**Table 3:** Clinical scores of UV-B treated latently infected B6 Mice.

Reactivation	Opacity	Neovascularization	Blink response
First	0.9 ± 0.5	1.6 ± 0.7	3.8 ± 0.7
Second	1.5 ± 0.5	2.6 ± 0.9	3.1 ± 0.6
Third	2.2 ± 0.5	3.6 ± 1.1	2.2 ± 0.6
Fourth	2.4 ± 0.5	4.4 ± 1.0	2.0 ± 0.6

**Table 4:** Clinical scores of UV-B treated uninfected B6 mice.

Reactivation	Opacity	Neovascularization	Blink response
First	0.4 ± 0.4	0.9 ± 0.7	4.4 ± 0.5
Second	0.4 ± 0.4	0.9 ± 0.7	4.4 ± 0.5
Third	0.6 ± 0.4	0.9 ± 0.7	4.3 ± 0.4
Fourth	0.5 ± 0.4	0.9 ± 0.7	4.2 ± 0.5

We latently infected mice and reactivated them initially at 5 weeks following infection. These same mice were then subsequently reactivated 4 weeks following the first reactivation (Second); then 4 weeks following that reactivation (Third) and finally 4 weeks after that reactivation (Fourth). The number of animals used in 3A was 20 and 10 for 3B.

Data is expressed as mean ± standard error of the mean.

Statistical differences between the first reactivated group and other groups were only achieved for the third reactivation (P < 0.05) and fourth reactivation group (P < 0.01).

**Figure 2:**
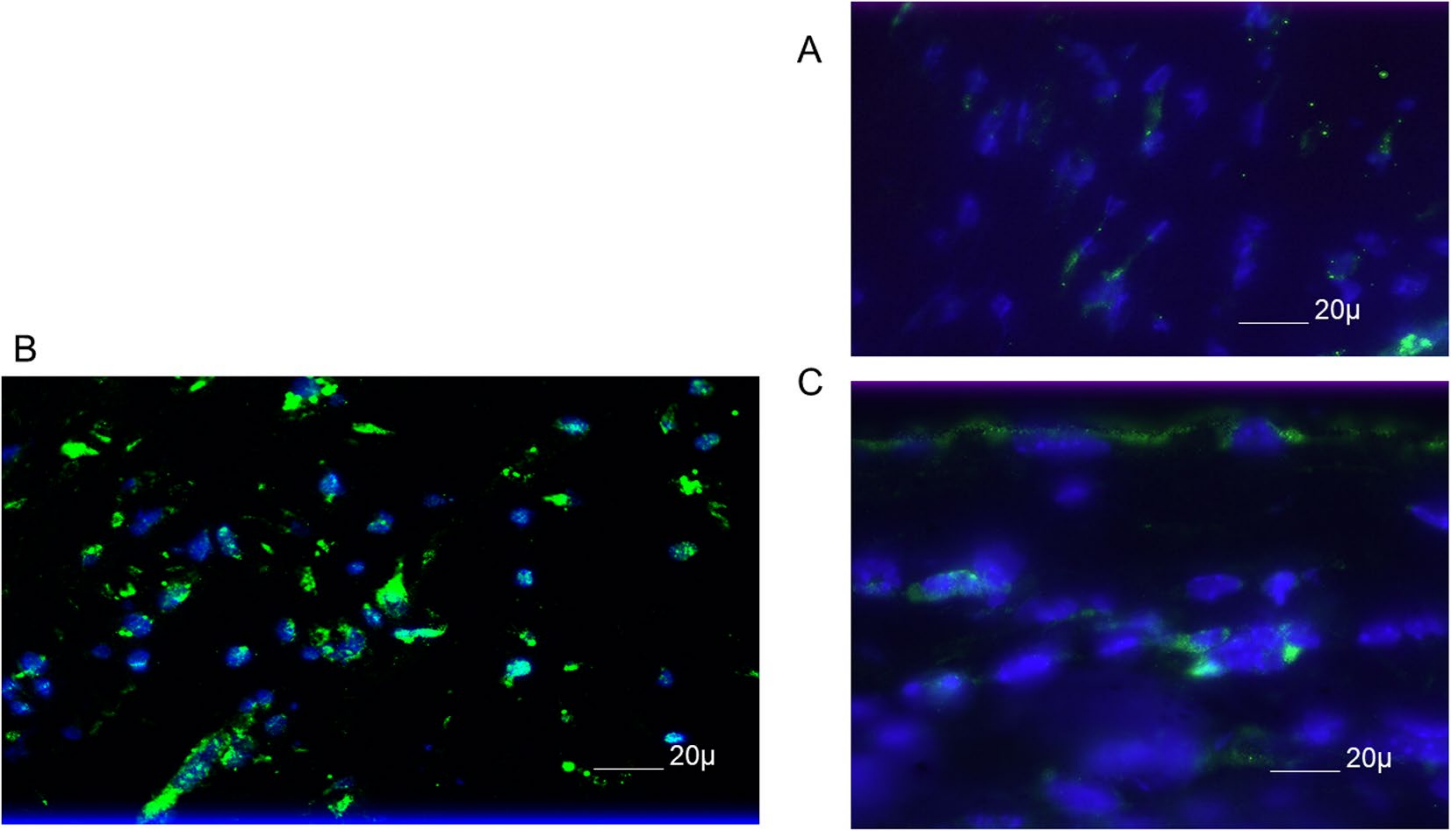
B6 mice latently infected with HSV-1 McKrae displayed greater expression of IFNα than either those latently infected mice not UV-B treated, or those wild-type mice treated with UV-B alone. Each of the groups shown consisted of 7 mice, and the results shown were representative for the animals in a particular group. The figure shown is from corneas obtained from UV-B only treated mice (**A**) and latently infected mice treated with UV-B (**B**) and sham treated (**C**) at 3 days post UV-B treatment. Similar results were noted at Day 2 and Day 5 post-UV-B treatment. Original magnification ×80. Bar, 20 µm.

To more fully understand the events that occur in the cornea following UV-B reactivation we evaluated the type I interferon response. Many other investigators have shown that type I IFN production is very important in resistance to primary infection^35,36^. However, little is known about such responses following UV-B reactivation. Consequently, we evaluated corneal sections at Days 1, 2, 3 and 5 following UV-B reactivation for the presence of IFNα. Representative results from Day 3 indicate that following reactivation there are a significant number of cells expressing IFNα found in reactivated corneas (Fig. 2B), but not found in corneas of latently infected, but not UV-B reactivated mice (Fig. 2A). Likewise, uninfected mice receiving UV-B treatment did not display many cells positive for IFNα expression (Fig. 2C). IFNα was not detected at Day 1 post-infection and Day 2 only had a few positive cells in the latently infected and reactivated group, which is why we chose to show Day 3 where differences were most apparent. These observations are similar to what we generally observe, as following reactivation there is no virus in tear film swabs detected at Day 1 following UV-B treatment and little virus at Day 2 following UV-B treatment^7^. As indicated in the figure legend, we did not detect significant IFNα expression by Day 5 post-reactivation.

**Figure 3:**
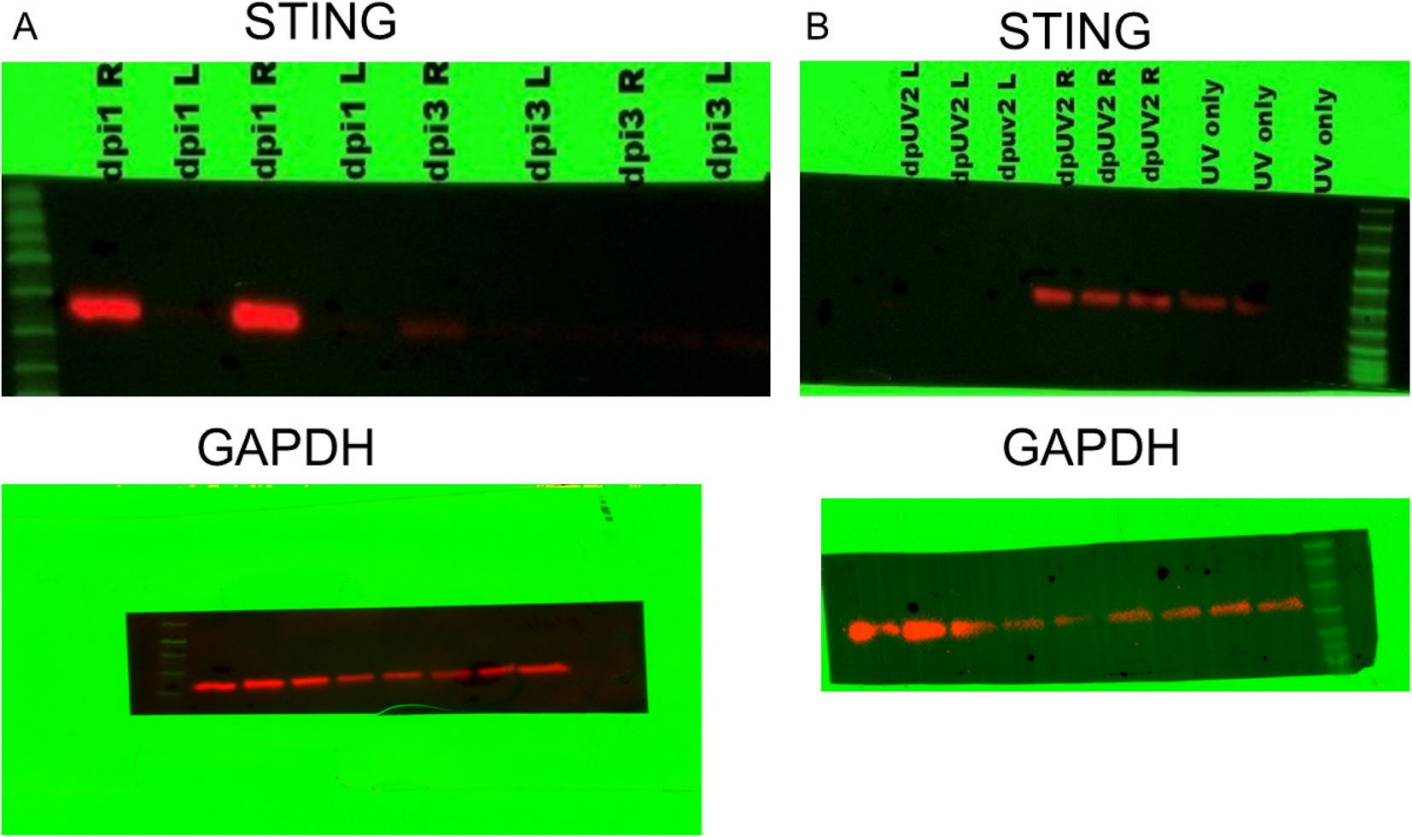
HSV-1 induces expression of STING. Each of the groups shown consisted of 7 mice, and the results shown were representative for the animals in a particular group. (**A**) B6 mice were infected with HSV-1 McKrae, and corneas were removed from either the infected eye (right) or the uninfected eye (left) and corneas removed at either 1 or 3 days post-infection. Protein was isolated from these eyes and subjected to Western Blot analysis. Figure shows that only infected eyes displayed STING expression and that by day 3 that expression was mostly gone. (**B**) Corneas were removed from latently infected 2 days after UV-B irradiation and protein was analyzed for STING expression. Figure shows that STING expression is only seen in latently infected eyes following UV-B treatment (right) and not in the contralateral eye (left). Corneas from mice receiving only UV-B treatment displayed low levels of STING expression. As a loading control the blot was stained with GAPDH antibody as seen in the bottom of (**A**) and (**B**). Similar data was observed from an independent study (data not shown).

We next evaluated STING expression following either primary infection with HSV-1 or following UV-B induced reactivation. We chose this pathway as this is one of the critical pathways that stimulates the innate immune response following infection^37,38^. We initially attempted to determine levels in phosphorylation of STING as a means of determining activation of this sensing molecule. However, we quickly found that such an analysis would be impossible due to the transient presence of phosphorylated STING that is seen in cultured cells^39^. However, when Western blots of primary infection were run, we noted that STING is expressed following infection (Fig. 3A), while STING expression in the uninfected eye could not be detected. Furthermore, STING expression is maximally expressed at Day 1 following infection and almost undetectable by day 3 post-infection. Thus, we decided to measure STING expression in latently infected mice following UV-B reactivation. Figure 3B indicates that there is expression of STING in latently infected mice undergoing UV-B treatment, while the contralateral eye did not demonstrate measurable STING. That said, this figure also demonstrated that UV-B treatment of uninfected corneas does result in STING expression in some, but not all mice. In contrast, latently infected mice undergoing UV-B treatment displayed levels of STING expression that are greater than in uninfected mice treated with UV-B (Fig. 3B).

**Figure 4:**
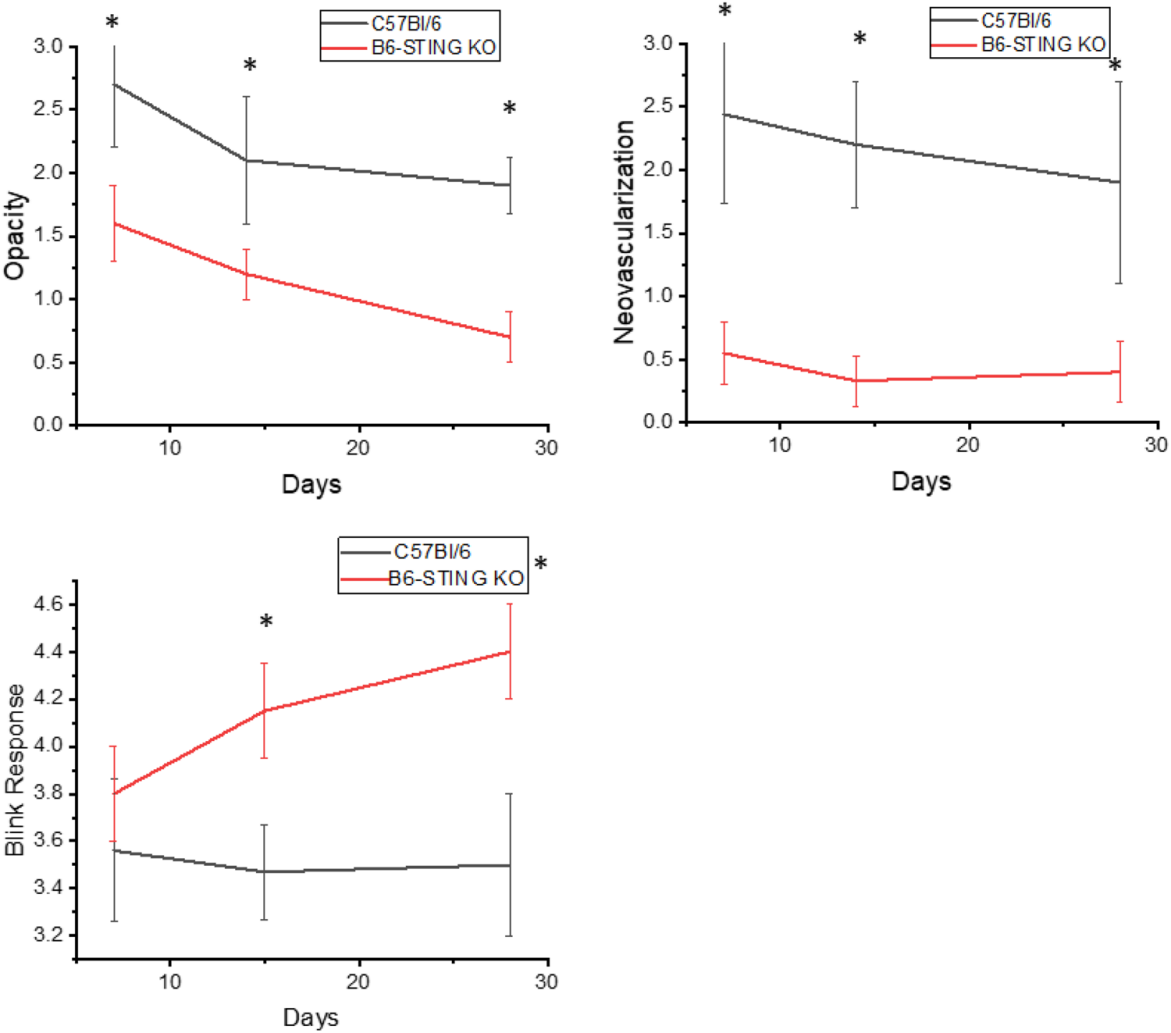
STING expression is required for corneal disease in a recurrent model of HSK. B6 (n = 12) and B6-STING KO (n = 18) mice were latently infected with HSV-1 McKrae at 10^5^ pfu/mouse. These mice were subjected to UV-B irradiation to induce viral reactivation from latency. Results indicated that wild-type B6 mice displayed significant corneal disease following UV-B reactivation while B6-STING mice did not develop significant corneal disease. Data represents mean ± SEM for these groups and significance was determined by rank sum analysis, asterisk represents p < 0.02–0.001 by Rank Sum analysis. Similar data was observed from 2 independent studies, though the group sizes were smaller (data not shown).

**Figure 5:**
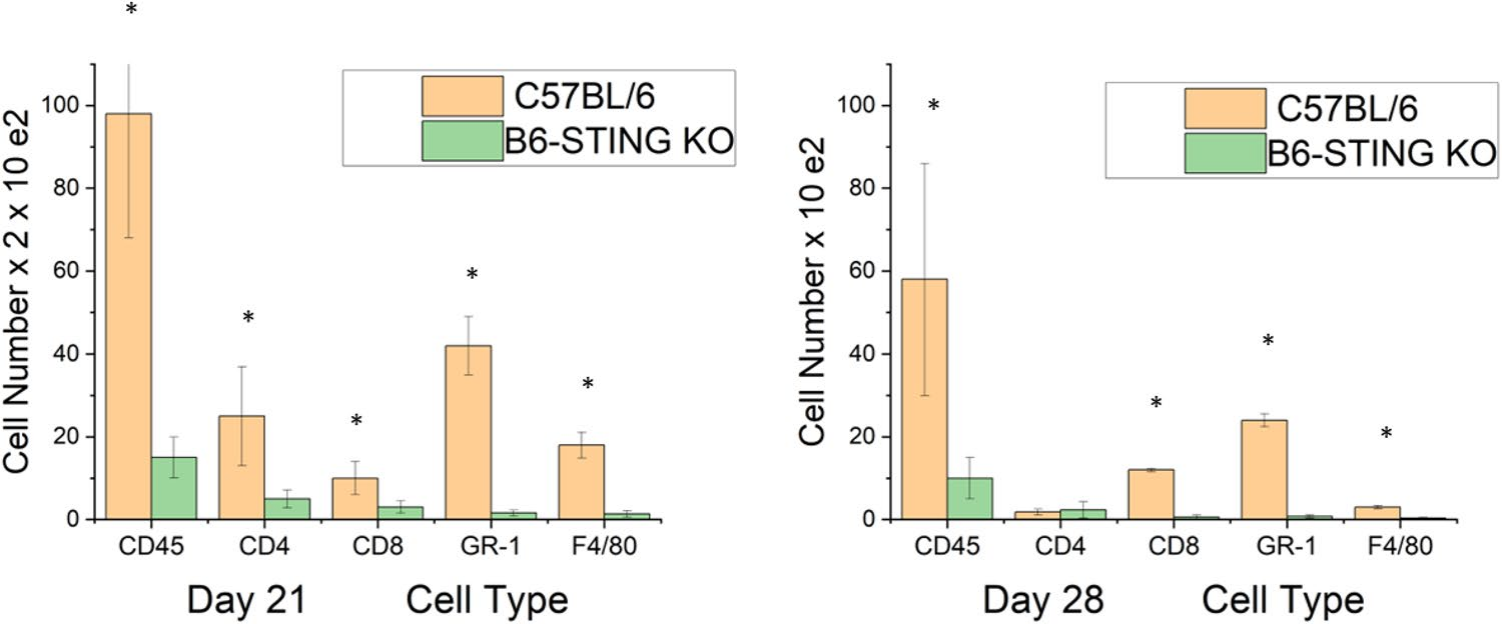
Flow cytometric analysis of corneas from wild-type displayed great numbers of inflammatory cells than corneas from B6-STING KO mice. We reactivated B6 (n = 15) and B6-STING KO mice (n = 15) and harvested their corneas on Day 21 and Day 28 following UV-B irradiation. Cells were subjected to flow cytometric analysis for the indicated markers. Results indicate that wild-type B6 mice had a greater total inflammatory infiltrate (CD45^+^ cells) and a corresponding increase in cell numbers for neutrophils (CD45^+^CD11B^+^F4/80^−^GR-1^+^), CD4^+^ T cells, CD8^+^ T cells and macrophages (CD45^+^CD11B^+^GR-1^−^F4/80^+^) at Day 21 post-reactivation and that a similar profile was seen at day 28 as well, though the number of cells were substantially reduced at this time point. Data represents mean ± SEM for individually analyzed corneas from these groups. Results were analyzed by student’s t-test and significance was determined for all cell types at Day 21 and all except CD4^+^ T cells at Day 28 (p < 0.05–0.01) (see asterisks). Similar data was observed from 2 independent studies, though the group sizes were smaller (data not shown).

This increase in STING expression seen in latently infected mice following UV-B treated mice allowed us to hypothesize that STING expression might also be related to corneal disease. It had been previously reported that mice with compromised STING expression displayed significantly greater mortality following HSV-1 infection^40,41^. Consequently, we decided to compare female B6-STING KO mice to female wild-type B6 mice in our reactivation model. We chose female mice for this analysis as they routinely display greater disease^8^. Our initial attempts to latently infect B6-STING KO mice underscored their increased sensitivity to HSV-1 infection^40,41^, in that mice infected with our normal dose of 10^6^ PFU HSV-1 McKrae displayed a 60% mortality rate even though they had been given anti-HSV-1 antibody at the time of infection. The wild-type B6 mice did not demonstrate any deaths following our standard means of establishing latency (Table 5). We included other gene targeted mice to accentuate the particular sensitivity of STING deficient mice to the normal protocol for establishing latency. We therefore infected B6 and B6-STING KO mice with 10^5^ PFU HSV-1 McKrae along with anti-HSV-1 antisera. Infecting with this lower dose of virus did not result in significant mortality for the B6-STING KO mice, this likely due to the dose dependency that is consistent with many different infectious agents. When these latently infected mice were subjected to UV-B exposure, the B6-STING KO mice were expected to display greater viral shedding but results only demonstrated only a slight and insignificant greater number of mice showing reactivated virus in their tear film at day 3 post reactivation (7/18) than that observed with wild-type B6 mice (4/12) infected at the same dose of virus. This was also reflected by the fact that the viral titers for those mice displaying infectious virus in their tear film were indistinguishable (345 ± 85 for B6-STING KO mice and 401 ± 94 for wild-type B6 mice). However, when corneal disease was evaluated, the B6-STING KO mice displayed significantly less corneal disease when compared to their wild-type counterparts did (Fig. 4). We next evaluated the inflammatory infiltrate in wild-type and B6-STING KO mice. This analysis demonstrated that the B6-STING KO mice displayed significantly fewer CD45^+^ cells (leukocytes) (Fig. 5). We next evaluated these CD45^+^ cells for T cell, neutrophil and macrophage subsets. Such an analysis indicated that the B6-STING KO mice had significantly fewer cells of all subsets at day 21 post-reactivation and significantly fewer CD8^+^T cells, GR-1^+^ (neutrophils) and F4/80^+^ (macrophages) cells at day 28 post-reactivation (Fig. 5). These data indicate that STING expression is required for mice to display significant recurrent HSK following UV-B induced reactivation.

**Table 5:** Strain response to infection to establish latency and reactivation.

Strain	Corneal disease^a^	Mortality^b^	Number of mice
C57BL/6	Normal	None	50
B6-CD4 KO	Little to none	None	30
B6-CD8 KO	High	None	30
B6-CD4/CD8 dbl KO	None	10%	15
B6-IFNγ KO	Normal	< 10%	30
B6-STING KO	Little to none	72%	25

^a^Refers to published results using these strains of mice. B6-CD4 KO, B6-CD4 KO and B6-CD4/CD8 dbl KO^42^; B6-IFNγ KO^43^.

^b^Mortality of mouse strain when infected with 1 × 10^6^ pfu HSV-1 McKrae strain in the presence of anti-HSV-1 antibodies.

## Discussion

Recurrent HSV-1 infection which results in HSK, is a leading cause of infectious blindness involving the cornea in the developed world^1–4^. Following primary corneal infection by HSV-1, the virus travels to the TG where it has the capacity to establish latency. This established latency can be disrupted by various stressors leading to reactivation of the virus and spread to peripheral tissues^1–4^. In humans, primary disease is typically without clinical symptoms^2–4^. Surveys indicate that most cases demonstrating clinical disease are a result of reactivation of a latent HSV-1 infection^2–4^. Disease is initiated by the presence reactivated virus in the cornea, which in turn, initiates an immunopathologic condition that results in corneal inflammation, which when severe, will result in chronic damage that might result in permanent damage to the cornea^2,4^. There are several animal models of this disease, mostly involving rabbits and mice as the animal of choice^8,9,13,15^. Our laboratory has been studying a mouse model of both acute infection and to a much greater extent, recurrent model of HSK that requires some sort of stimulation that reactivates the virus from latency in the TG^8,21,42–44^. While we were using the UV-B induced model originally described by Shimeld et al.^27,34^, we decided several years ago to compare that means of inducing reactivation with using CP, which had also been shown to reactivate an active infection from latency^45,46^. We decided to restrict this comparison to CP as opposed to other means of HSV-1 reactivation, such as hyperthermia and chemical treatments such as sodium butyrate^20–25^ as UV-B and CP are both related to changes in the the immune response following treatment^33,47^. This comparison confirmed that UV-B induced reactivation was superior to CP induced reactivation as UV-B treatment not only leads to latently infected cells in the TG releasing infectious virus, but also results in corneal disease, but CP treatment did not lead to corneal disease. This underscores the immunosuppressive nature of CP treatment^33^ and at the same time restricts it’s use as a method to establish a recurrent model of HSK.

In addition to demonstrating that UV-B irradiation was superior to CP, we really wanted to better understand the levels of reactivation from UV-B irradiation. It had always been our suspicion that that tear film data was, at best, an underestimation of the true reactivation rate following UV-B induced reactivation. This hypothesis was strongly supported from our recent publication indicating that most, if not all, corneas possessed HSV-1 antigen following UV-B reactivation^31^. Nonetheless, one could argue that there might have been residual antigen in corneas remaining from the primary infection at the time of reactivation. To convince ourselves that our hypothesis was accurate, we removed corneas from mice 3 days post-reactivation and made extracts that were then tested for infectious virus. We clearly show that the great majority of latently infected mice treated with UV-B contained infectious virus while less than ten percent of latently infected mice who were not subjected to UV-B treatment contained infectious virus. These studies revealed the interesting observation that there was a tendency of female mice to demonstrate a somewhat greater percentage of mice with infectious virus and higher mean titers of virus than seen in similarly treated male corneas from latently infected mice undergoing UV-B induced reactivation. Though, upon statistical analysis, only the mean viral titers for female mice proved to be significant. While we can only speculate of this observation, one could interpret these results to be a partial explanation for why we see greater disease in female vs. male mice in our reactivation model^8^. It is also interesting to note that the range of viral titers for female corneas with infectious virus was very broad ranging from 8 to 6800 pfu (Table 2). This could be viewed as indicating that disease is most severe in those bearing the greatest viral load. However, a recent report by Drayman et al.^48^ indicates that immune activation is exclusively restricted to cells that are undergoing unproductive infection rather than productive infection. This report further suggests that cells that are producing infectious virus are not capable of stimulating a significant innate immune response due to the effectiveness of infectious virus to control such responses in these cells. Consequently, the activation of an immune response to the virus may not be driven by the total number of productively infected cells, but rather the number of non-productively infected cells. This in part may explain the difficulty of detecting ICP4 message in reactivated corneas. It is known that ICP4 is required for expression viral genes that produce infectious virus^34^. Interestingly, most UV-B-reactivated mice express some level of disease, while those latently infected and not UV-B-reactivated nor those corneas from uninfected mice undergoing UV-B irradiation alone do not display remarkable disease.

Another concept that we decided to examine was the hypothesis that serial reactivation would result in progressively increased disease in mice. Our data supports that is the case. We believe that this is potentially the result of two, non-mutually exclusive mechanisms. The first would be that serial reactivation would act as a booster for the pathogenic CD4^+^ T cells that mediate corneal disease^42,49^. The second would be the result of changes in nerve fiber regrowth into the cornea following infection^49^ and reactivation^31^. We have recently published that such regrowth leads to increased disease due to the preferential regrowth of sympathetic vs sensory nerves, which predisposes the cornea to increased inflammation and that CD4^+^ T cells are required for that to occur^31^. Future studies could be performed to evaluate the relative contribution of these factors to increased disease. Furthermore, this observation suggests that controlling reactivation occurrences is very important in preventing serious complications from recurrent HSK.

That concept led us to investigate surrogate markers to determine the extent of immune activation following UV-B reactivation in cells of the cornea. The two markers we chose to investigate were IFNα and STING. Analysis of corneas following reactivation of virus indicated that exposure to UV-B irradiation alone will stimulate a few cells to express IFNα, but that all corneas from latently infected mice that were UV-B-reactivated demonstrated apparently more cells expressing IFNα. This, we believe is an indication that the corneas of latently infected mice are responding to the presence of new virus following reactivation leading to increased expression of IFNα in the cells of the cornea^26^. We next monitored the expression of the known pattern recognition molecule STING that is activated following HSV-1 infection^27,34,35^. The cGAS-cGAMP-STING pathway recognizes cytoplasmic DNA and recruits and activates the kinases TBK1 and IKKβ resulting in the activation of IRF3 and NF-κB, which culminates in the production of pro-inflammatory cytokines and type I IFNs^50–54^. Initially, we attempted to distinguish phosphorylated STING from unphosphorylated STING as a means of detecting activated STING. This proved to be impossible in vivo when using corneas from these mice, probably due to the transient nature of phosphorylated STING and the difficulty of detecting phosphorylated STING in the presence of murine corneas. However, we noted that the latently infected mice following UV-B induced reactivation expressed more STING protein as determined by Western blot analysis than either corneas from un-reactivated and latently infected mice or corneas from uninfected mice receiving UV-B treatment alone. It should be noted that we demonstrated that mice undergoing primary infection also expressed significantly greater levels of STING than corneas from uninfected mice, which was not detectable in uninfected mice. Thus, our data demonstrates that the presence of HSV-1, whether via primary infection or the result of UV-B induced reactivation, stimulates STING protein expression in these infected corneas. We were somewhat surprised that infection/reactivation induces STING expression, as the virus produces at least two different proteins that interfere with the STING pathway of innate immune activation. This has been shown by data indicating that viruses lacking VP22 also have a more robust cGAMP activity, which indicates an interaction between VP22 and cGAS that reduces cGAMP production^54^. Thus, VP22 from HSV-1 acts as an antagonist of IFN-β activation by the cGAS-STING pathway by colocalizing and inhibiting the enzymatic activity of cGAS^54^. In addition, ICP27 is a multifunctional immediate early tegument protein essential for HSV-1 replication and is conserved among all herpesviruses. ICP27 functions in transporting mRNA transcripts from the nucleus to the cytosol^55^. Nevertheless, ICP27 also can inhibit innate immunity^56^ by counteracting the production of type I IFNs. ICP27 was shown to interfere with the production of type I IFN by interacting with STING and TBK1 and reducing the phosphorylation of IRF3^57^. Christensen et al.^55^ suggested a model for this inhibition whereby ICP27 has low affinity for inactivated TBK1 but higher affinity to TBK1 in the TBK1-STING complex leading to an interaction with both factors^57^. Thus HSV-1 invests some of its genetic capital to directly target this pathway. Nonetheless, our data clearly indicates that HSV-1 infection/reactivation results in an induction of STING production.

We finally went on to determine the impact of STING expression on HSK. As described earlier in this report, the STING pathway is a major factor in the cell’s ability to recognize the presence of unwanted DNA in the cytoplasm^50–54^. It had also been reported that in the absence of STING, mice were highly susceptible to infections by HSV-1^37,38^. These reports indicate that this pathway is critical in developing an innate immune response to HSV-1. We confirm this increased susceptibility of B6-STING KO mice in that even the presence of human anti-HSV-1 did not prevent significant numbers of mice dying from our normal dosage of 10^6^ PFU, which rarely leads to death of wild-type B6 mice. This led to our using a dose of 10^5^ PFU to infect these and wild-type mice for these studies. It should be noted that when using this lower dose there were no differences in viral reactivation rates or viral titers following UV-B reactivation. Since it is accepted that adaptive immune responses are preceded by a strong innate immune response to a particular antigen, we hypothesized that STING played a significant role in the development of a T cell response to HSV-1. This would lead one to postulate that since CD4^+^T cells play a central role in the development of HSK^58–64^, mice that possess an impaired STING pathway would also not display significant corneal disease. The results demonstrating significantly less CD4^+^T cells at Day 21 in B6-STNG mice support the notion that lack of STING expression results in significantly fewer CD4^+^ T cells, which might be due to suboptimal expansion of CD4^+^ T cells resulting from impaired innate immunity. Furthermore, reduced CD4^+^ T cell infiltration and activation will impair the presence of other inflammatory cells which characterize HSK, these include, though are not limited to macrophages and neutrophils. Neutrophils are of particular note as they tend to predominate the inflammatory infiltrate and are known to be a major cause of corneal damage during this disease^60,61,63^. Thus, lack of STING signaling leads to the reduced disease phenotype as the presence of inflammatory cells in the corneas of STING-targeted mice are reduced across the board when compared to animals with an intact STING signaling pathway. This further emphasizes the importance of innate immune responses to presence of HSV-1 to stimulate a CD4^+^ T cell response to the virus that can drive the immunopathology that typifies HSK. While it could be argued that lack of STING could result in significantly greater infection of the trigeminal ganglia, we discount that as being improbable for two reasons. The first being that similar studies in which infectivity of the TG was measured using B6-CD28 KO^65^ and B6-CD137 KO mice^30^, while at first demonstrating greater genome copies and LAT+ cells during the first 21 days post infection, levels normalized to wild-type levels at times corresponding to when mice would be reactivated. Thus, indicating that the level of TG infection at the time of reactivation would be the same for wild-type and immunocompromised animals. The reason for this has not been determined, but likely involves both virological and immunological factors. Secondly, the fact that shedding of virus was indistinguishable between B6 and B6-STING KO mice also argues that possible minor differences in TG infection does not have any impact on virus being released at the periphery, which is the proximate cause of recurrent HSK seen in these animals. Finally, it should be noted that B6-STING KO mice when first characterized for leukocyte levels, there was no indication that these mice were impaired in their ability to produce leukocytes, only that their responses to cyclic dinucleotides was compromised by lack of STING expression^66^.

## Data Availability

The datasets used and/or analyzed during the current study available from the corresponding author on reasonable request.

## References

[1] Tuli S, Sonal S, Yanoff JS, Duker M, Wiggs JL, Miller D, Azar DT, Goldstein MH, Rosen ES, Duker JS, Rao NA, Augsburger JJ, Sadum AA, Shuman JS, Diamond GR, Dutton JJ (2009). Herpes simplex keratitis. Ophthalmology.

[2] Farooq AV, Shukla D (2012). Herpes simplex epithelial and stromal keratitis: An epidemiologic update. Surv. Ophthalmol..

[3] Rowe AM, St Leger AJ, Jeon S, Dhaliwal DK, Knickelbein JE, Hendricks RL (2013). Herpes keratitis. Prog. Retinal Eye Res..

[4] Azher TN, Yin XT, Tajifirouz D, Huang AJ, Stuart PM (2017). Herpes simplex keratitis: Challenges in diagnosis and clinical management. Clin. Ophthalmol..

[5] Preston CM (2000). Repression of viral transcription during herpes simplex virus latency. J. Gen. Virol..

[6] Summers BC, Margolis TP, Leib DA (2001). Herpes simplex virus type 1 corneal infection results in periocular disease by zosteriform spread. J. Virol..

[7] Miller JK, Laycock KA, Umphress JA, Hook KK, Stuart PM, Pepose JS (1996). A comparison of recurrent and primary herpes simplex keratitis in NIH inbred mice. Cornea..

[8] Morris J, Stuart PM, Rogge M, Potter C, Gupta N, Yin XT (2012). Recurrent herpetic stromal keratitis in mice, a model for studying human HSK. J. Vis. Exp..

[9] BenMohamed L, Osorio N, Srivastava R, Khan AA, Simpson JL, Wechsler SL (2015). Decreased reactivation of a herpes simplex virus type 1 (HSV-1) latency-associated transcript (LAT) mutant using the in vivo mouse UV-B model of induced reactivation. J. Neurovirol..

[10] West DM, Del Rosso CR, Yin XT, Stuart PM (2014). CXCL1 but not IL-6 is required for recurrent herpetic stromal keratitis. J. Immunol..

[11] Ledbetter EC, da Silva EC, Kim SG, Dubovi EJ, Schwark WS (2012). Frequency of spontaneous canine herpesvirus-1 reactivation and ocular viral shedding in latently infected dogs and canine herpesvirus-1 reactivation and ocular viral shedding induced by topical administration of cyclosporine and systemic administration of corticosteroids. Am. J. Vet. Res..

[12] Ledbetter EC, Kice NC, Matusow RB, Dubovi EJ, Kim SG (2010). The effect of topical ocular corticosteroid administration in dogs with experimentally induced latent canine herpesvirus-1 infection. Exp. Eye Res..

[13] Perng GC, Ghiasi H, Slanina SM, Nesburn AB, Wechsler SL (1996). The Spontaneous reactivation function of the herpes simplex virus type 1 LAT gene resides completely within the first 1.5 kilobases of the 8.3-kilobase primary transcript. J. Virol..

[14] Beyer CF, Arens MQ, Hill JM, Rose BT, Hill GA, Lin DT (1989). Penetrating keratoplasty in rabbits induces latent HSV-1 reactivation when corticosteroids are used. Curr. Eye Res..

[15] Shimomura Y, Dudley JB, Gangarosa LP, Hill JM (1985). HSV-1 quantitation from rabbit neural tissues after epinephrine induced reactivation. Investig. Ophthalmol. Vis. Sci..

[16] Gordon YJ, Romanowski E, Berman J, Vikoren P, Lin LS, Schlessinger D, Araullo-Cruz T (1990). Vanadate promotes reactivation and iontophoresis-induced ocular shedding of latent HSV-1 W in different host animals. Curr. Eye Res..

[17] Green MT, Rosborough JP, Dunkel EC (1981). In vivo reactivation of herpes simplex virus in rabbit trigeminal ganglia: Electrode model. Infect. Immun..

[18] Gordon YJ, Araullo-Cruz TP, Romanowski E (1986). The development of an improved murine iontophoresis reactivation model for the study of H5V–1 latency. Investig. Ophthalmol. Vis. Sci..

[19] Gordon YJ, Romanowski E, Araullo-Cruz T (1990). A fast, simple reactivation method for the study of HSV-1 latency in the rabbit ocular model. Investig. Ophthalmol. Vis. Sci..

[20] Harwick J, Romanowski E, Araullo-Cruz (1987). Timolol promotes reactivation of latent HSV-1 in the mouse iontophoresis model. Investig. Ophthalmol. Vis. Sci..

[21] Stuart, P. M. & Keadle, T. L. Recurrent herpetic stromal keratitis in mice: A model for studying human HSK. *Clin. Dev. Immunol*. 2012, 728480 (2012). 10.1155/2012/728480 PMC3347728 22593769

[22] Khan AA, Srivastava R, Chentoufi AA, Garg S, Yu DC, Vahed H, Huang L, Syed SA, Furness JN, Tran TT, Anthony NB, Mclaren CE, Sidney J, Sette A, Noelle RJ, BenMohamad L (2017). Bolstering the number and function of HSV-1–specific CD8^+^ effector memory T cells and tissue-resident memory T cells in latently infected trigeminal ganglia reduces recurrent ocular herpes infection and disease. J. Immunol..

[23] Nesburn AB, Elliott JH, Leibowitz HM (1967). Spontaneous reactivation of experimental herpes simplex keratitis in rabbits. Arch. Ophthalmol..

[24] Sawtell NM, Thompson RL (1992). Rapid in vivo reactivation of herpes simplex virus in latently infected murine ganglionic neurons after transient hyperthermia. J. Virol..

[25] Webre JM, Hill JM, Nolan NM (2012). Rabbit and mouse models of HSV-1 latency, reactivation, and recurrent eye diseases. J. Biomed. Biotechnol..

[26] Shimeld C, Hill TJ, Blyth B, Easty D (1989). An improved model of recurrent herpetic 681 eye disease in mice. Curr. Eye Res..

[27] Shimeld C, Hill TJ, Blyth WA, Easty DL (1990). Passive immunization protects the 683 mouse eye from damage after herpes simplex virus infection by limiting spread of 684 virus in the nervous system. J. Gen. Virol..

[28] Xia L, Zhang S, Cao Z, Hu Y, Yang H, Wang D (2013). Interleukin-17 enhanced immunoinflammatory lesions in a mouse model of recurrent herpetic keratitis. Microbes Infect..

[29] Yin D, Ling S, Wang D (2021). Targeting herpes simplex virus with CRISPR–Cas9 cures herpetic stromal keratitis in mice. Nat. Biotechnol..

[30] Yin XT, Baugnon NK, Krishnan R, Potter CA, Yarlagadda S, Keadle TL, Stuart PM (2022). CD137 costimulation is associated with reduced herpetic stromal keratitis and with developing normal CD8^+^ T cells in trigeminal ganglia. J. Gen. Virol..

[31] Yun H, Yin XT, Stuart PM, St. Leger AJ (2022). Sensory nerve retraction and sympathetic nerve innervation contribute to immunopathology of murine recurrent Herpes Stromal Keratitis (HSK). Investig. Ophthal. Vis. Sci..

[32] Kurata T, Kurata K, Aoyama Y (1978). Reactivation of herpes simplex virus (type 2) infection in trigeminal ganglia and oral lips with cyclophosphamide treatment. Jpn. J. Exp. Med..

[33] Hadjis AD, Nunes NS, Khan SM, Fletcher RE, Pohl AdP, Venzon DJ, Eckhaus MA, Kanakry CG (2022). Post transplantation cyclophosphamide uniquely restrains alloreactive CD4^+^T cell proliferation and differentiation after MHC-haploidentical hematopoietic cell transplantation. Front. Immunol..

[34] Knickelbein JE, Khanna KM, Yee MB, Baty CJ, Kinchington PR, Hendricks RL (2008). Noncytotoxic lytic granule-mediated CD8+ T cell inhibition of HSV-1 reactivation from neuronal latency. Science.

[35] Conrady CD, Jones H, Zheng M, Carr DJJ (2011). A functional Type I interferon pathway drives resistance to cornea herpes simplex virus Type 1 infection by recruitment of leukocytes. J. Biomed. Res..

[36] Conrady C, Zheng M, Mandal N, van Rooijen N, Carr D (2013). IFN-α-driven CCL2 production recruits inflammatory monocytes to infection site in mice. Mucosal Immunol..

[37] Conrady CD, Zheng M, Fitzgerald KA, Liu C, Carr DJJ (2012). Resistance to HSV-1 infection in the epithelium resides with the novel innate sensor, IFI-16. Mucosal Immunol..

[38] Royer DJ, Carr DJJ (2016). A STING-dependent innate sensing pathway mediates resistance to corneal HSV-1 infection via upregulation of the antiviral effector tetherin. Mucosal Immunol..

[39] Calvet-Mirabent M, Claiborne DT, Deruaz M, Tanno S, Serra C, Delgado-Arévalo C, Sánchez-Cerrillo I, de Los SI, Sanz J, García-Fraile L, Sánchez-Madrid F, Alfranca A, Muñoz-Fernández MÁ, Allen TM, Buzón MJ, Balazs A, Vrbanac V, Martín-Gayo E (2022). Poly I: C and STING agonist-primed DC increase lymphoid tissue polyfunctional HIV-1-specific CD8+ T cells and limit CD4+ T-cell loss in BLT mice. Eur. J. Immunol..

[40] Parker ZM, Murphy AA, Leib DA (2015). Role of the DNA sensor STING in protection from lethal infection following corneal and intracerebral challenge with herpes simplex virus 1. J. Virol..

[41] Ceron S, North BJ, Taylor SA, Leib DA (2019). The STING agonist 5,6-dimethylxanthenone-4-acetic acid (DMXAA) stimulates an antiviral state and protects mice against herpes simplex virus-induced neurological disease. Virology.

[42] Keadle TL, Morris JE, Pepose JS, Stuart PM (2002). CD4^+^ and CD8^+^ cells are key participants in the development of recurrent herpetic stromal keratitis in mice. Microb. Pathog..

[43] Stuart PM, Sumers B, Morris JE, Morrison LA, Leib DA (2004). CD8^+^ T cells control corneal disease following ocular infection with herpes simplex virus type 1. J. Gen. Virol..

[44] Keadle TL, Alexander DE, Leib DA, Stuart PM (2008). Interferon gamma is not required for recurrent stromal keratitis. Virology.

[45] Kurata T, Kurata K, Aoyama Y (1976). Reactivation of herpes simplex virus (type 2) infection in trigeminal ganglia and oral lips with cyclophosphamide treatment. Jpn. J. Exp. Med..

[46] Wrzos H, Rapp F (1985). Experimental model for activation of genital herpes simplex virus. J. Infect. Dis..

[47] Tse BCY, Ferguson AL, Koay YC, Grau GE, Don AS, Byrne SN (2023). Exposure to solar ultraviolet radiation establishes a novel immune suppressive lipidome in skin-draining lymph nodes. Front. Immunol..

[48] Drayman N, Patel P, Vistain L, Tay S (2019). HSV-1 single-cell analysis reveals the activation of anti-viral and developmental programs in distinct sub-populations. Elife.

[49] Yun H, Yee MB, Lathrop KL, Kinchington PR, St. Leger AJ (2020). Production of the cytokine VEGF-A by CD4^+^ T and myeloid cells disrupts the corneal nerve landscape and promotes herpes stromal keratitis. Immunity.

[50] Burdette DL, Vance RE (2013). STING and the innate immune response to nucleic acids in the cytosol. Nat. Immunol..

[51] Barber GN (2011). Innate immune DNA sensing pathways: STING, AIMII and the regulation of interferon production and inflammatory responses. Curr. Opin. Immunol..

[52] Motwani M, Pesiridis S, Fitzgerald KA (2019). DNA sensing by the cGAS-STING pathway in health and disease. Nat. Rev. Genet..

[53] Carty M, Guy C, Bowie AG (2021). Detection of viral infections by innate immunity. Biochem. Pharm..

[54] Huang J, You H, Su C, Li Y, Chen S, Zheng C (2018). Herpes simplex virus 1 tegument protein VP22 abrogates cGAS/STING-mediated antiviral innate immunity. J. Virol..

[55] Sandri-Goldin RM (2011). The many roles of the highly interactive HSV protein ICP27, a key regulator of infection. Future Microbiol..

[56] Kolb AW, Lee K, Larsen I, Craven M, Brandt CR (2016). Quantitative trait locus based virulence determinant mapping of the HSV-1 genome in murine ocular infection: Genes involved in viral regulatory and innate immune networks contribute to virulence. PLoS Pathog..

[57] Christensen MH, Jensen SB, Miettinen JJ, Luecke S, Prabakaran T, Reinert LS, Mettenleiter T, Chen ZJ, Knipe DM, Sandri-Goldin RM, Enquist LW, Hartmann R, Mogensen TH, Rice SA, Nyman TA, Matikainen S, Paludan SR (2016). HSV-1 ICP27 targets the TBK1-activated STING signalsome to inhibit virus-induced type I IFN expression. EMBO J..

[58] Tumpey TM, Cheng H, Yan XT, Oakes JE, Lausch RN (1998). Chemokine synthesis in the HSV-1-infected cornea and its suppression by interleukin-10. J. Leuk. Biol..

[59] Babu JS, Kanagat S, Rouse BT (1995). T cell cytokine mRNA expression during the course of the immunopathologic ocular disease herpetic stromal keratitis. J. Immunol..

[60] Thomas J, Gangappa S, Kanagat S, Rouse BT (1997). On the essential involvement of neutrophils in the immunopathologic disease: Herpetic stromal keratitis. J. Immunol..

[61] Divito SJ, Hendricks RL (2008). Activated inflammatory infiltrate in HSV-1-infected corneas without herpes stromal keratitis. Investig. Ophthalmol. Vis. Sci..

[62] Stuart PM, Morris JE, Sidhu M, Keadle TL (2008). CCL3 protects mice from corneal pathology during recurrent HSV-1 infection. Front. Biosci..

[63] West DM, Del Rosso CR, Yin XT, Stuart PM (2014). CXCL1 but not IL-6 is required for recurrent herpetic stromal keratitis. J. Immunol..

[64] Tajifirouz D, West DM, Yin XT, Potter CA, Klein RS, Stuart PM (2017). CXCL9 compensates for the absence of CXCL10 during recurrent herpetic stromal keratitis. Virology.

[65] Yin XT, Baugnon NK, Potter CA, Tai S, Keadle TL, Stuart PM (2019). CD28 costimulation is required for development of herpetic stromal keratitis but does not prevent establishment of latency. J. Virol..

[66] Sauer JD, Sotelo-Troha K, von Moltke J, Monroe KM, Rae CS, Brubaker SW, Hyodo M, Hayakama Y, Woodward JJ, Portnoy DA, Vance RE (2011). The N-ethyl-N-nitrosourea-induced *Goldenticket* mouse mutant reveals an essential function of Sting in the in vivo interferon response to *Listeria monocytogenes* and cyclic dinucleotides. Infect. Immun..

